# Correction: NF-κB regulated expression of A20 controls IKK dependent repression of RIPK1 induced cell death in activated T cells

**DOI:** 10.1038/s41418-024-01424-0

**Published:** 2024-12-03

**Authors:** Scott Layzell, Alessandro Barbarulo, Geert van Loo, Rudi Beyaert, Benedict Seddon

**Affiliations:** 1 Institute of Immunity and Transplantation, Division of Infection and Immunity, University College London, The Pears Building, Hampstead, London, UK; 2 VIB-UGent Center for Inflammation Research, UGent Department for Biomedical Molecular Biology, Unit of Molecular Signal Transduction in Inflammation, Gent, Belgium

**Keywords:** Immune cell death, T cells, Cell death and immune response

Correction to: *Cell Death & Differentiation* 10.1038/s41418-024-01383-6, published online 26 September 2024

In this article the author’s name Scott Layzell was incorrectly written as “By Scott Layzell”.

The original article has been corrected.

