# Supplementary material for: NF-κB regulated expression of A20 controls IKK dependent repression of RIPK1 induced cell death in activated T cells
Source: Cell Death Differ. 2024 Sep 26;32(2):256–70. doi: 10.1038/s41418-024-01383-6 (PMC11802744; doi:10.1038/s41418-024-01383-6)
Supplement: Supplementary file 1 — Supplementary figures [file 41418_2024_1383_MOESM1_ESM.pdf]

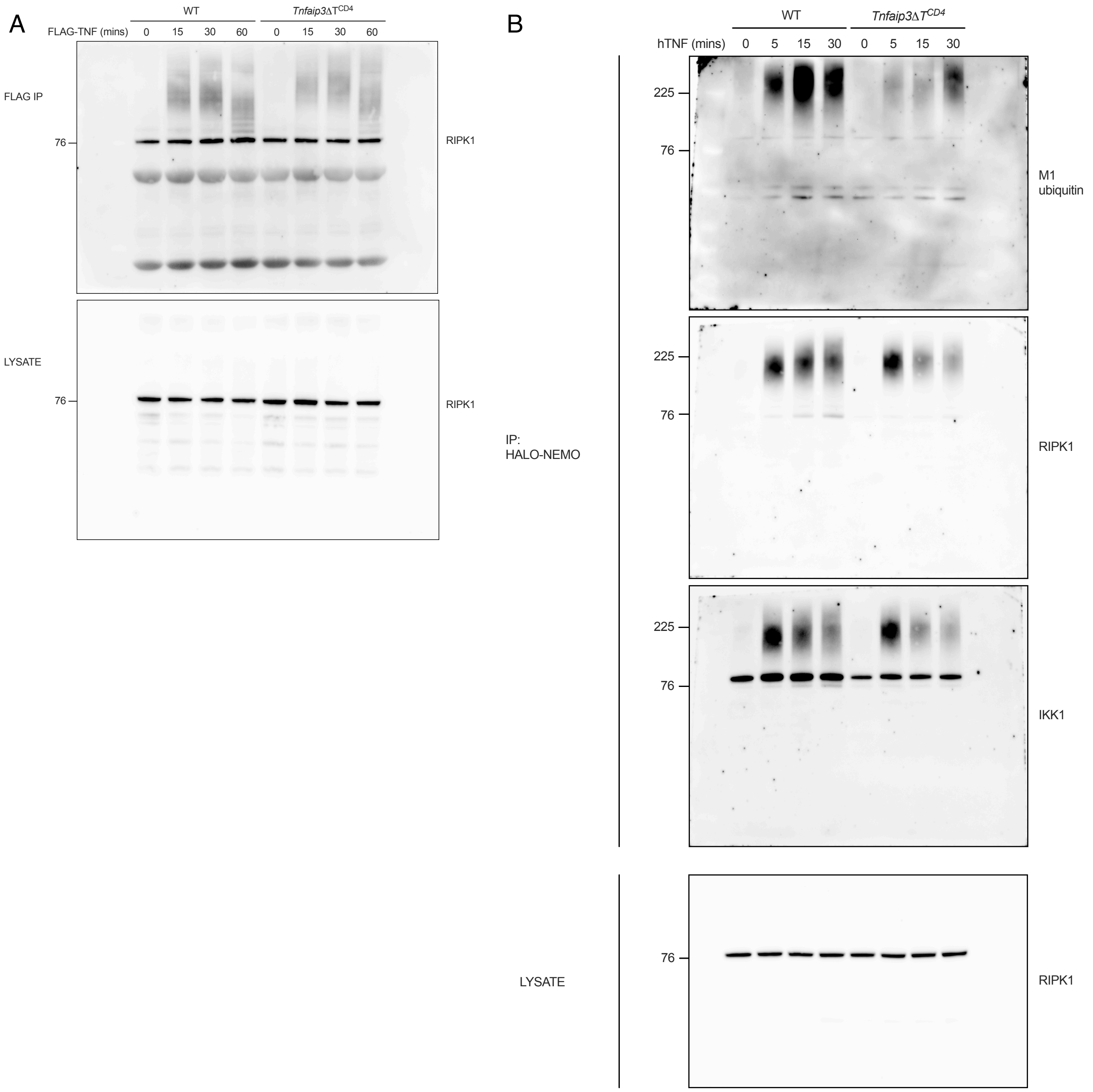

supplementary **Figure 1** - Defective ubiquitin scaffold formation in complex I in *Tnfaip3* deficient T cells. Uncropped images of immuoblots from figure 5B. Thymocytes from Tnfaip3ΔT^{CD4} and Cre –ve litter mates were stimulated with (A) FLAG-TNF or (B) hTNF for different times, lysed and immunoprecipitated with either anti-FLAG-sepharose or HALO-NEMO beads and analysed by immunoblotting for RIPK1, M1 ubiquitin and IKK1. Data are representative of 3 independent experiments. Uncropped

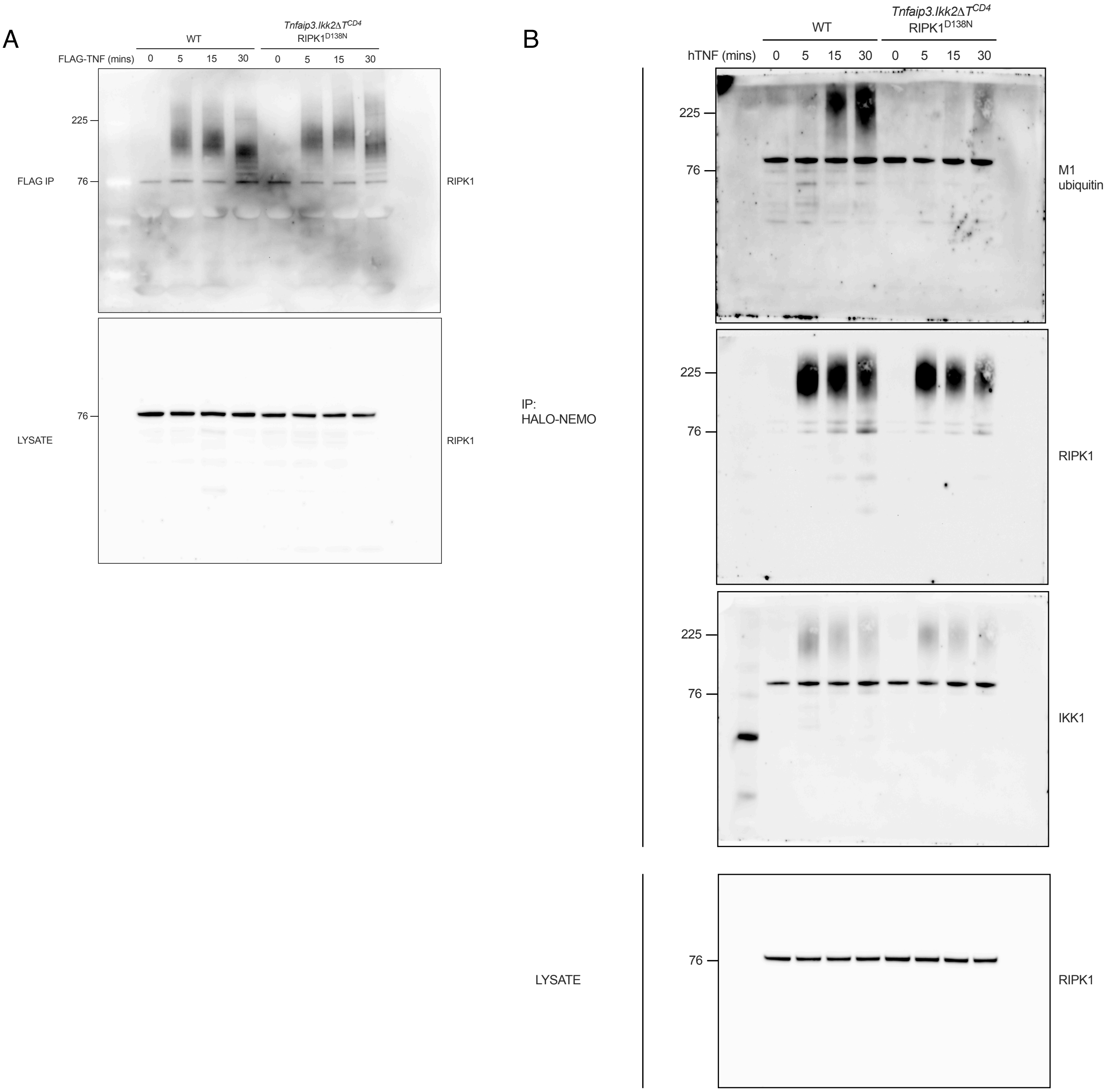

supplementary **Figure 2 - Defective ubiquitin scaffold formation in complex I in *Ikk2.Tnfaip3* deficient T cells.** Thymocytes from *Tnfaip3.Ikk2*ΔT^{CD4} RIPK1^{D138N} and Cre –ve litter mates were stimulated with (A) FLAG-TNF or (B) hTNF for different times, lysed and immunoprecipitated with either anti-FLAG-sepharose or HALO-NEMO beads and analysed by immunoblotting for RIPK1, M1 ubiquitin and IKK1. Data are representative of 3 independent experiments.
